# Systematic literature review to identify templates for reporting pre-hospital major incident medical management

**DOI:** 10.1186/1757-7241-21-S1-S18

**Published:** 2013-05-28

**Authors:** S Fattah, M Rehn, E Reierth, T Wisborg

**Affiliations:** 1 Department of Research and Development, Norwegian Air Ambulance Foundation, Norway; 2 Anaesthesia and Critical Care Research Group, Faculty of Health Sciences, University of Tromsø, Norway; 3 Department of Anaesthesia and Intensive Care, Akershus University Hospital, Norway; 4 Science and Health Library, University of Tromsø, Norway; 5 Department of Anaesthesiology and Intensive Care, Hammerfest Hospital, Norway

## Background

Pre-hospital major incident medical management may be improved through the collection and analysis of standardized data. Improvements in this field have been advocated in the previous years. This study was designed to identify templates reporting such management.

## Methods

A systematic review was conducted according to the PRISMA guidelines. The protocol for the study was registered in PROSPERO and published in BMJ Open (1). In the search strategy the first set of entry terms describes major incidents published during the last 20 years. The second set of entry terms describes templates for collecting data from such incidents. Predefined free search phases were combined with the first two sets. A modified search strategy was used for the grey literature. The articles that were included were subjected to quality analysis. Reference lists of included literature were hand searched.

## Results

Of the 8497 articles identified in the main database search, 8389 were excluded based on their titles and abstracts. Another 96 items were excluded based on full-text articles, as they did not meet the inclusion criteria. The remaining 12 were included. In the grey literature all 107 articles were excluded. Reference lists of the included literature identified five articles. One relevant article was identified by chance after completion of the search. In the total of 18 included articles 10 different templates or sets of data are described; two methodologies for assessing major incident response, three templates intended for reporting from exercises, two guidelines for reporting in medical journals, two analyses of previous disasters, and one Utstein-Style template.

## Conclusions

Each of the above-mentioned templates includes some data regarding the pre-hospital medical management of major incidents. However, none of them were specifically designed for this purpose. In order to allow rapid dissemination of areas for improvement, there is a need for a field-friendly template that is especially focused on such management.
